# Hypoplastic Left Heart Syndrome: A Case Report

**DOI:** 10.31729/jnma.8243

**Published:** 2023-08-31

**Authors:** Sunil Raja Manandhar, Anjila Ghimire, Dipendra Rai, Sabina Shrestha, Pritha Adhikari

**Affiliations:** 1 Neonatology Unit, Department of Paediatrics, Kathmandu Medical College and Teaching Hospital, Sinamangal, Kathmandu, Nepal

**Keywords:** *case report*, *congenital heart disease*, *hypoplastic left heart syndrome*

## Abstract

Hypoplastic left heart syndrome is a rare critical congenital heart defect constituting a prevalence of 1/3,500 to 1/12,500 live births in which there is obstructions to the blood flow within the left heart. Here we present a case of a full-term 38 weeks male baby with a birth weight of 3.5 kg, and no obvious physical deformity referred to our centre at 22 hours of life for respiratory distress and cyanosis. At 23 hours of life, he was diagnosed with hypoplastic left heart syndrome and kept on Prostaglandin E1 infusion till the 12th day of life. The baby had a large ventricular septal defect and atrial septal defect with a severely hypoplastic left ventricle, hypoplastic aortic root, ascending aorta and post-ductal coarctation of the aorta. The diagnosis was reconfirmed by computed tomography cardiac angiography on the 10th day of life with the same cardiac findings suggestive of hypoplastic left heart syndrome.

## INTRODUCTION

Hypoplastic left heart syndrome (HLHS) is a lethal congenital heart defect constituting a prevalence of 1/3,500 to 1/12,500 live births.^1^ The incidence depends on the definition, but it has been reported to be 8 to 25 per 100,000 liveborn neonates without selection during pregnancy.^2,3^ HLHS includes various lesions with a dominant right ventricle (RV) and systemic outflow obstruction including various shunt lesions e.g. ventricular septal defect (VSD) and atrial septal defect (ASD).^4^ Underdevelopment of the left-sided structures of the heart may involve aortic valve atresia, atresia or stenosis of the mitral valve and hypoplasia of the ascending aorta and aortic arch.^5^ We present a case of a full-term, 38 weeks male baby diagnosed with HLHS at 23 hours of life by bedside echocardiography.

## CASE REPORT

**Figure 1:**
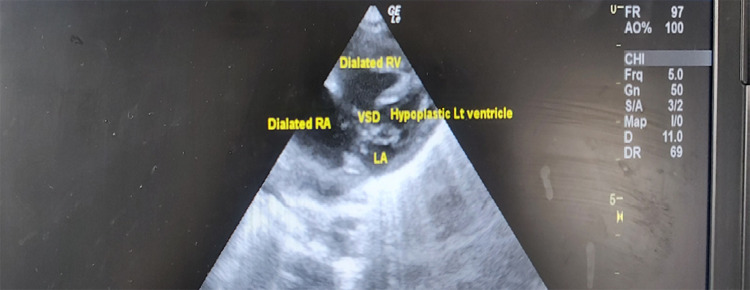
2D echocardiography subcostal view showing dilated RV and RA, large VSD with hypoplastic LV at 23 hours of life.

A full-term, male baby with a birth weight of 3.5 kg was delivered via elective cesarean section at a tertiary hospital in Kathmandu Valley. The baby was borne by non-consanguineous parents with a maternal obstetric history of G_3_P_1_L_1_A_1_ without obvious physical deformity. The baby developed respiratory distress with cyanosis at 20 hours of life and was referred to our centre for neonatal intensive care. A lethargic, cyanosed, hypotensive baby was received at 22 hours of life at emergency with pre-ductal saturation of partial pressure of oxygen (SPO_2_) was 65%. The baby was intubated with initial resuscitation and kept under a mechanical ventilator after shifting to the neonatal intensive care unit (NICU) of KMCTH. Despite under mechanical ventilation, the baby's saturation did not improve and bedside echocardiography was done at 23 hours of life which revealed a hugely dilated right ventricle (RV), dilated right atrium (RA), severely hypoplastic left ventricle (LV), 10 mm membranous ventricular septal defect (VSD) (bi-directional), 5 mm atrial septal defect (ASD) (bi-directional), hypoplastic ascending aorta, small patent ductus arteriosus (PDA) 1.2 mm with post-ductal coarctation (Figure 1).

**Figure 2:**
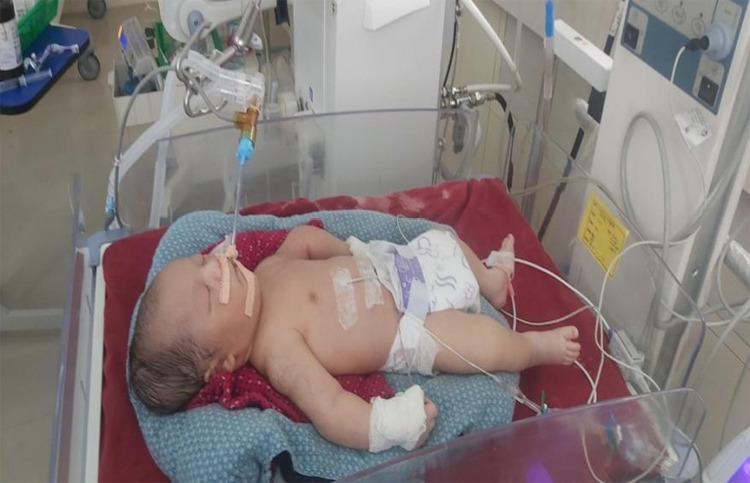
The baby was under a mechanical ventilator on the third day of life, showing no obvious external visible physical deformity.

**Figure 3:**
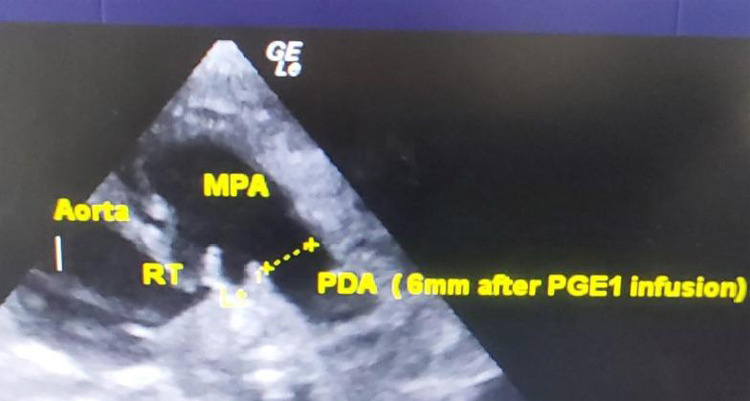
2D echocardiography parasternal short axis view showing hypoplastic aorta root, dilated MPA and large PDA after PGE1 infusion at 4 days of life.

**Figure 4:**
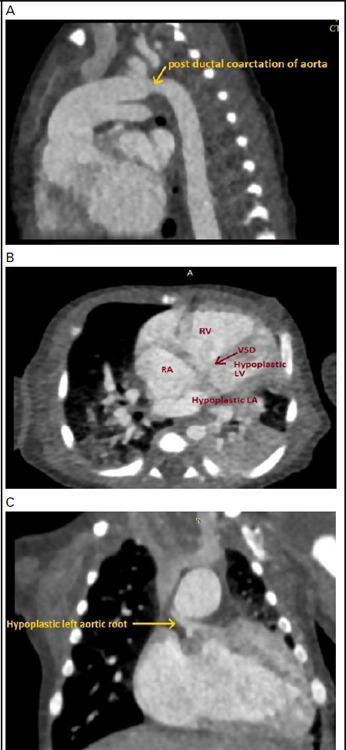
CT cardiac angio showing A) Post ductal coarctation of the aorta, B) hypoplastic left ventricle, dilated right ventricle and ventricular septal defect, C) hypoplastic left-sided ascending aortic root.

Due to the persistent rise of serum creatinine (2.5 mg/dl), cardiac computerized tomography (CT) angiography was done only on the 10^th^ day of life, however, PGE1 infusion was continued for 25 hrs of life. Cardiac CT with the study of the aorta and pulmonary arteries showed large VSD, ASD, dilated RA, Dilated RV with the severely hypoplastic left ventricle, hypoplastic left atrium, hypoplastic aortic root with the ascending aorta, left-sided aortic arch and post ductal coarctation of the aorta. (Figure 4).

The baby was referred to the cardiac surgery centre for the operative procedure on the 12^th^ day of life but, unfortunately, the baby could not survive.

## DISCUSSION

The left-sided heart structures are underdeveloped as a result of the complex aetiology of HLHS. The causes of this defect can be divided into two categories: obstruction of flow into the left ventricle (mitral valve atresia or stenosis, restrictive foramen ovale), or obstruction of flow out of the left ventricle (obstruction of the left ventricular outflow tract). Aortic stenosis-related obstruction to left ventricular (LV) outflow during fetal development raises LV afterload and results in LV hypertrophy, which eventually causes LV dilatation and reduced LV contractility. Furthermore, decreased blood flow through the LV hinders the ventricle's growth, resulting in hypoplasia. The neonates with HLHS are reliant on a patent ductus arteriosus (PDA) for blood supply to the coronary arteries and systemic circulation. To keep the ductal patency after birth, prostaglandin E1 (PGE1) must be continuously infused. In contrast to the normal heart's series circulation, these patients are thought to have systemic and pulmonary circulations operating simultaneously.^6^

As this is one of the rare critical congenital heart diseases with severe forms of cardiac malformation, very less case notes have been reported so far. An autopsy case published in Sao Paulo, Brazil highlighted that HLHS is a lethal congenital heart disease and despite the availability of improved management and surgical treatment in developed countries, it is still associated with high mortality, especially in the early neonatal period and before the second stage of reconstruction surgery.^7^

The HLHS, which is characterized anatomically by an insufficiently sized left ventricle to support the systemic circulation and varying degrees of valvular obstructions, involves a spectrum of blood flow obstructions inside the left heart. The main problems mentioned include hypoplasia of the aortic arch and ascending aorta, as well as atresia or stenosis of the mitral and aortic valves. Additionally, an ascending aorta diameter of less than 3 mm is substantially related to aortic coarctation.^8^ Similar to this, in our case also, the baby had a hypoplastic left ventricle, hypoplastic aortic arch and ascending aorta along with large VSD and ASD.

As HLHS is one of the critical mixed-flow congenital heart diseases with various cardiac malformations, it is very much difficult to save these types of babies, particularly in low-middle-income countries like Nepal where neonatal cardiac surgical infrastructure is suboptimal.
